# Examining the Impact of Selected Sociodemographic Factors and Cancer-Related Fatalistic Beliefs on Patient Engagement via Health Information Technology Among Older Adults: Cross-Sectional Analysis

**DOI:** 10.2196/44777

**Published:** 2023-10-20

**Authors:** Maryum Zaidi, Priscilla Gazarian, Heather Mattie, Lisa Kennedy Sheldon, C Ann Gakumo

**Affiliations:** 1 Solomont School of Nursing Zuckerberg College of Health Sciences University of Massachusetts Lowell Lowell, MA United States; 2 Manning College of Nursing & Health Sciences University of Massachusetts Boston Boston, MA United States; 3 Harvard T H Chan School of Public Health Boston, MA United States; 4 St Joseph Hospital New Hampshire, NH United States; 5 College of Nursing University of Cincinnati Cincinnati, OH United States

**Keywords:** health information technology, patient portals, older adults, digital health, self-management, mobile phone

## Abstract

**Background:**

Despite the role of health information technology (HIT) in patient engagement processes and government incentives for HIT development, research regarding HIT is lacking among older adults with a high burden of chronic diseases such as cancer. This study examines the role of selected sociodemographic factors and cancer-related fatalistic beliefs on patient engagement expressed through HIT use for patient engagement in adults aged ≥65 years. We controlled for cancer diagnosis to account for its potential influence on patient engagement.

**Objective:**

This study has 2 aims: to investigate the role of sociodemographic factors such as race, education, poverty index, and psychosocial factors of cancer fatalistic beliefs in accessing and using HIT in older adults and to examine the association between access and use of HIT in the self-management domain of patient activation that serves as a precursor to patient engagement.

**Methods:**

This is a secondary data analysis of a subset of the Health Information National Trend Survey (Health Information National Trend Survey 4, cycle 3). The subset included individuals aged ≥65 years with and without a cancer diagnosis. The relationships between access to and use of HIT to several sociodemographic variables and psychosocial factors of fatalistic beliefs were analyzed. Logistic and linear regression models were fit to study these associations.

**Results:**

This study included 180 individuals aged ≥65 years with a cancer diagnosis and 398 without a diagnosis. This analysis indicated that having less than a college education level (*P*=<.001), being an individual from an ethnic and minority group (*P*=<.001), and living in poverty (*P*=.001) were significantly associated with decreased access to HIT. Reduced HIT use was associated with less than a college education (*P*=.001) and poverty(*P*=.02). This analysis also indicated that fatalistic beliefs about cancer were significantly associated with lower HIT use (*P*=.03). Specifically, a 1-point increase in the cancer fatalistic belief score was associated with a 36% decrease in HIT use. We found that controlling for cancer diagnosis did not affect the outcomes for sociodemographic variables or fatalistic beliefs about cancer. However, patients with access to HIT had a self-management domain of patient activation (SMD) score of 0.21 points higher (*P*=.003) compared with patients who did not have access. SMD score was higher by 0.28 points (*P*=.002) for individuals who used HIT and 0.14 points higher (*P*=.04) who had a prior diagnosis of cancer.

**Conclusions:**

Sociodemographic factors (education, race, poverty, and cancer fatalistic beliefs) impact HIT access and use in older adults, regardless of prior cancer diagnosis. Among older adults, HIT users report higher self-management, which is essential for patient activation and engagement**.**

## Introduction

### Background

Patient engagement (PE) has gained prominence as a major component of achieving key performance indicators in health care [1-3]. PE in health care decision-making has become an expectation worldwide but lacks planning, design, and precision in specific medical settings and populations, specifically older adults [4,5]. Engagement is described as a cognitive and emotional state expressed through observable behaviors [6]. As a process, PE can go through significant stages: engaging, staying engaged, disengaging, and reengaging [7]. Health information technology (HIT) is a powerful yet underutilized tool for PE across many medical specialties [8-12]. HIT is a broad term encompassing an array of technologies referring to electronic health records, personal health records, patient portals, secure access to email providers, or requesting electronic prescribing [13-15] to collect, store, share, and analyze health information [16]. HIT has shown promising results in improving the quality of life and self-management of people with multiple chronic illnesses [17]. The prevalence of chronic diseases among older adults is considerably higher, with almost 95% having at least 1 and approximately 80% experiencing 2 or more such conditions [18].

Despite the emphasis and government spending on HIT and its role in PE, critical discussions regarding its access and use among older adults are insufficient. Evidence suggests that using HIT to communicate with providers is not always discussed during medical consultations [19]. Furthermore, in most cases, HIT access has been discussed in previously engaged patients [20]. Therefore, digital inclusion emerges as a social determinant of health when specific populations, including older adults, face barriers owing to limited access, non-English language availability, or insufficient knowledge to use digital technology [21]. Older adults are part of a growing and racially diverse group in the United States [22]. Despite being late adopters of technology, their use of the internet has been on the rise. From a reported 14% in 2000, it skyrocketed to 64% in 2016, and more recently reached 75% [23,24]. Nevertheless, there is a lack of comprehensive understanding of the impact of HIT access and use on self-management and engagement processes in older adults [25-27].

Cancer is a chronic disease that requires patients to engage with a health care team over time to discuss different treatment options [28]. PE in cancer care delivery results in higher quality of care, greater patient satisfaction, and improved cost containment [29-31]. Although further research is needed on the use of HIT among older adults diagnosed with cancer, it has been observed that these patients tend to demonstrate higher levels of activation and engagement in their health care. This can be attributed to the existing body of literature that underscores the advantages of HIT and the supportive role played by oncology health care providers [32-36]. Therefore, to investigate the influence of HIT use in older adults, we examined specific sociodemographic and psychosocial factors of fatalistic beliefs while accounting for the potential impact of a cancer diagnosis, which might affect their level of engagement.

In this analysis, we used selected demographic variables such as age, sex, race, education level, household income, and poverty level on the access and use of patient portals in older adults. All these variables are associated with HIT access and use in adults [37-39]; however, these variables have been limited to older adults, where the burden of chronic disease is high [40]. Furthermore, the association between HIT use and PE in older adults has not been previously assessed. Consequently, we introduced an additional objective to examine the relationship between HIT use and the self-management domain (SMD) of patient activation, which is a precursor to PE [41,42].

Accessing HIT entails using tools to access health information. It describes passive, one-way information access and can replace or enhance in-person interaction with the health care system or provider [43]. Use refers to actions taken after access to HIT to generate knowledge to engage in health care [44]. In addition, PE is a phenomenon that is deeply psychological and results from the cognitive, emotional, and behavioral endorsement of individuals toward their health care [6]. Therefore, in addition to focusing on sociodemographic factors, we examined fatalistic beliefs about cancer regarding access and use of HIT. Cancer fatalism refers to a belief or attitude that cancer is an unavoidable and inevitable disease and that there is little or nothing individuals can do to prevent it or improve their chances of survival if diagnosed. This fatalistic perspective may lead the general public to dismiss the importance of adopting healthy behaviors and participating in preventive screenings [45-48]. The impact of these beliefs on the use of HIT tools for PE is unknown; hence, we included cancer fatalism in our analysis.

Finally, we included an SMD for patient activation. Patient activation, as developed by Hibbard et al [49], refers to an individual’s knowledge, skills, and confidence in managing their own health and health care especially in older adults [49,50]. The concept of patient activation was found to have 5 domains: self-management, collaboration with the provider, maintenance of health functions, prevention of decline, and access to appropriate and high-quality care. We used the SMD, which refers to behaviors associated with taking action to manage and engage in one’s care and is negatively associated with fatalism about one’s health [49]. Recent evidence indicates that self-management and patient activation can be supported by the use of HIT [51]; however, this relationship has not yet been examined in older adults. Patients with a diagnosis of cancer are reported to have 70% higher odds of being activated [32,35], hence we controlled for the effect of cancer diagnosis.

### Specific Aims

Below are our specific aims and hypotheses.

#### Aim 1

We aim to examine the relationship between selected patient-specific sociodemographic (education, income, and race) and psychosocial factors (fatalistic beliefs) on access to and use of HIT in individuals when controlled for cancer diagnosis:

- Hypothesis 1-a: we hypothesize that a stronger association exists between older adults’ education and access to and use of HIT in individuals when controlled for cancer diagnosis.
- Hypothesis 1-b: we hypothesize that there is a stronger association between access and use of HIT among White individuals and people of color when controlled for cancer diagnosis.
- Hypothesis 1-c: we hypothesize that there is a stronger association between older adults’ income and access to and use of HIT in individuals when controlled for cancer diagnosis.
- Hypothesis 1-d: we hypothesized that having more fatalistic beliefs about cancer will negatively affect access to and use of HIT in individuals when controlled for cancer diagnosis.

#### Aim 2

We aim to examine the relationship between access to and use of HIT and the SMD score of patient activation measures in older adults, when controlled for cancer diagnosis:

- Hypothesis 2-a: low access to and use of the HIT will result in lower scores of the SMD of patient activation measure in individuals controlling for cancer diagnosis.

## Methods

### Data Source

This study is based on the Health Information National Trends Survey (HINTS), a dynamic resource for studying consumer engagement in health communication research [52]. Since its first cycle in 2003, a total of 14 cycles have addressed different health communication topics. HINTS 4 cycle 3 is the only cycle that has a measure for the SMD of patient activation, one of the main outcome variables for this proposed study, and is a precursor to the PE process. Therefore, we used HINTS 4 cycles 3 for this analysis.

In the original data set, the target population was adults aged 18 years or older in the civilian noninstitutionalized population of the United States. The third of 4 cycles, cycle 3, was conducted from September 2013 to December 2013. One respondent per household was selected for this cycle. The adults were selected by asking those with the next birthday to complete the survey. The Spanish questionnaire was also included in the package. The sampling frame consisted of a database of all nonvacant addresses used by the Marketing System Group to provide random samples of addresses. The sampling frames of the addresses were grouped into 3 explicit sampling strata. These groups consisted of addresses in areas with a high concentration of racial minority populations, areas with low concentrations of racial minority populations, and addresses located in counties comprising Central Appalachia regardless of the racial minority population.

### Study Design

The cross-sectional analysis for this study was limited to a subset of the original data set and included participants aged ≥65 years. The total number of individuals over 65 years of age with a diagnosis of cancer was 261 (180 after accounting for the missingness of variables used in the analyses) and without diagnosis was 604 (398 after accounting for the missingness of variables used in the analyses). HINTS 4 cycle 3 data were weighted using jackknife variance estimation to produce a representative sample of the US population [53].

### Measurements

#### Overview

First, access to HIT and use of HIT were dependent variables, and the independent variables were education, race, poverty level, income level, and fatalistic beliefs about cancer. For the second aim, we used access to and use of HIT as independent variables and the SMD of patient activation as a dependent variable to predict the effect of access to and use of HIT on SMD.

#### Access to HIT

In this study, access to the HIT variable was determined if an individual had access to the internet and knowledge about their provider maintaining electronic medical records. Access to the HIT was measured by combining 2 items, B1 and E1. The first variable, B1, asked individuals if they had access to the internet, and the second variable, E1, asked if they knew if their physician maintained their medical information in a computerized system. Access was given a score of 1 if both variables were affirmative and a score of 0 if one or both variables were reported to be absent. These 2 variables were combined to form a dichotomous variable that describes an individual’s access to HIT as a yes or no response.

#### Use of HIT

In this study, the use of the HIT was quantified by creating a score variable by combining answers to 6 questions in the survey. One point was given for affirmative responses to item B5 (g), “In the last 12 months, have you used the internet to keep track of personal health information such as care received, test results, or upcoming appointments?”; item B5 (h), “In the last 12 months, have you used email or the Internet to communicate with a doctor or a doctor’s office?”; and 4 items in B6, “In the past 12 months, have you used any of the following to exchange medical information with a health care professional: a. email, b. text message, c. an app on a smartphone or mobile device, d. video conference.” Owing to the minimal number of patients indicating text and app use, we dichotomized the use variable. If any individual used any of the communication channels with a provider in the last 12 months, they had a value of 1 for utility, and if they did not use these channels, they had a value of 0. These items were selected based on the use of the term HIT in the literature [54,55].

#### The Self-Activation Domain of Patient Activation

The domain of self-management for patient activation was operationalized using 6 questions from HINTS, adapted from the work by Hibbard et al [49]. In this study, these 6 items indicate that the patient has the confidence and ability to obtain the desired information about treatment or therapy. These questions were item D3 of the survey. One point each was given to the affirmative responses to questions, “In general, how often do you take with you a list of questions or concerns; take a list of all their prescribed medicines to the doctor; ask the doctor to explain a test, treatment, or procedure to them in detail; read information about new prescriptions, such as side effects and precautions; do research on a health and medical topic after seeing their doctor, and take with them any kind of health information they have found during doctor visits.” This resulted in scores ranging from 0 to 6, with 0 indicating the lowest level of self-management and 6 the highest level. Our use of these items was consistent with their previous use [55].

#### Sociodemographic Variables

Sociodemographic variables included race, education level, and income. Race was coded as a binary variable (White vs person of color), whereas education level and income were coded as categorical variables. Education level was used as a proxy for health literacy because a significant number of participants were missing observations related to health literacy items in the survey, and education had a high correlation with the items used to quantify health literacy. Education was divided into 3 categories, and income was divided into 4 categories. See Table 1 for the details of each category. In addition to conducting our analysis with income categories, we created a poverty variable by combining household income and the US poverty index from 2013 to (ASPE 2013 Poverty Guidelines, office of the assistance secretary for planning and evaluation [56]) run our analysis with both income and poverty index.

**Table 1:** Descriptive statistics of sample (N=578)^a^.

					With a diagnosis of cancer (n=180)		Without a diagnosis of cancer (n=398)
**Outcomes (engagement process)**							
	**Utility of HIT^b^, n (%)**						
		Yes		45 (25)		75 (18.8)	
		No		135 (75)		323 (81.2)	
	**Access to HIT, n (%)**						
		Yes		96 (53.3)		192 (48.2)	
		No		84 (46.7)		206 (51.8)	
	Self-management domain score, mean (SD)				2.737 (0.6)		2.608 (0.7)
	**Sociodemographics, n (%)**						
		**Race and ethnicity**					
			White	157 (87.2)		306 (76.9)	
			People of color	23 (12.8)		92 (23.1)	
	**Socioeconomic, n (%)**						
		**Education**					
			<12 y	22 (12.2)		48 (12.1)	
			12 y or completed high school	65 (36.1)		160 (40.2)	
			Post high school training and colleg*e*	93 (51.7)		190 (47.7)	
		**Income (US $)**					
			0-14,999	32 (17.8)		84 (21.1)	
			15,000-49,999	73 (40.6)		185 (46.5)	
			50,000-99,999	58 (32.2)		91 (22.9)	
			>99,000	17 (9.4)		38 (9.5)	
		**Poverty**					
			Yes	78 (43.3)		216 (54.3)	
			No	102 (56.7)		182 (45.7)	
**Psychosocial** **, mean (SD)**							
	Fatalistic beliefs				2.36 (0.60)		2.53 (0.62)

^a^Values are n (%) for binary and categorical variables and mean (SD) for continuous variables.

^b^HIT: health information technology.

#### Cancer Fatalistic Belief

Cancer fatalistic beliefs were operationalized with questions in items M5 (a, b, c, and e): “It seems like everything causes cancer,” “There is not much you can do to lower your chances of getting cancer,” “There are so many different recommendations about preventing cancer, it’s hard to know which ones to follow,” and “When I think about cancer, I automatically think about death.” These questions have been used in several previous studies to determine fatalism [47,57,58]. The last item, “When I think about cancer, I automatically think of death,” was new to this survey. All items were pretested with cognitive interviews and included in a national pilot test of 172 adults to ensure content validity before being included in the HINTS survey [59]. The answers to these questions ranged from 1 to 4 (from strongly agree to strongly disagree). For this analysis, the items were combined to yield a score for fatalistic beliefs.

### Statistical Analyses

We fit logistic regression and linear regression models in 2 independent samples of older adults: those with and those without a diagnosis of cancer using Stata (version 15; StataCorp). This analysis was run on weighted data to generalize the results to the entire US population. We included interaction terms to test each hypothesis and examine whether there is a difference among individuals who have had a diagnosis of cancer compared with those who have not.

### Ethical Consideration

This study was granted exempt status by the Institutional Review Board at the University of Massachusetts Boston, as the data used in this research is publicly accessible and has been de-identified per guideline(s): 45 CFR 46.104(d)(4) for secondary research for which consent is not required.

## Results

### Aim 1

To examine the relationship between selected patient-specific sociodemographic (education, income, and race) and psychosocial factors (fatalistic beliefs) on access to and use of HIT in individuals when controlled for cancer diagnosis.

#### Hypothesis 1-a

We hypothesized that a stronger association exists between older adults’ education and access to and use of HIT in individuals when controlled for cancer diagnosis.

Education level was used as a proxy for health literacy because a significant number of participants had missing observations related to health literacy items in the survey, and education had a high correlation with the items used to quantify health literacy. Two separate logistic regression models were used to analyze the association between education level and access to and use of HIT for cancer diagnosis. For access, individuals with an education level higher than college level or above were 7.52 (95% CI 3.66-15.48; *P*<.001) times more likely to have access to HIT, whereas those with a high school diploma or post high school training were 1.93 times (95% CI 1.005-3.70; *P*=.048) more likely to use HIT, compared with those with less than 12 years of schooling (Table 2). For the use of HIT, this analysis showed that higher education levels were associated with higher odds of using HIT. Those who had a college education or above were 3.43 (95% CI 1.32-8.9; *P*=.001) times more likely to use HIT, whereas those with a high school diploma or post–high school training were 1.33 (95% CI 0.54-3.3; *P*=.53) times more likely to use HIT, compared with those with less than 12 years of schooling. The result was statistically significant at the α =.05 level for individuals with at least a high school education for access to HIT and for those with a college education or higher for use of HIT. Hence, this hypothesis was supported in this analysis. There was no significant difference in access to (*P*=.28) or use of HIT (*P*=.20) in individuals when controlled for cancer diagnosis.

**Table 2:** Results of univariate models controlling for education.

Predictor			Access to HIT^a^				Use of HIT		
			OR^b^ (95% CI)		*P* value		OR (95% CI)		*P* value
Cancer			2.13 (0.53-8.4)		.28		1.03 (0.802-2.73)		.20
**Education**									
	Reference (<12 years of schooling)	1		N/A^c^		1		N/A	
	High school or after high school	1.93 (1.005-3.70)		.048		1.33 (0.54-3.3)		.53	
	Some college and graduate	7.5 (3.66-15.48)		<.001		3.43 (1.32-8.9)		.001	

^a^HIT: health information technology.

^b^OR: odds ratio.

^c^N/A: not applicable.

#### Hypothesis 1-b

We hypothesize that there is a stronger association between access and use of HIT among White individuals and people of color when controlled for cancer diagnosis.

Similar to the models mentioned above, we fit 2 logistic regression models to analyze the association between race and access and use of HIT, controlling for cancer diagnosis. Our sample size of people of color was small. Only 12.8% (23/180) of the individuals with a cancer diagnosis and 23.1% (92/398) of those without a cancer diagnosis were people of color. Due to the limited number of people of color in the sample, we did not stratify the individuals by race.

For access, compared with people of color, individuals who identified as White were 2.47 (95% CI 1.51-4.05; *P*<.001) times more likely to have access to HIT, whereas there was no significant difference about their use of HIT (*P*=.68). Hence, this hypothesis was supported for access to HIT but not for the use of HIT in this analysis. There was no significant difference in access to (*P*=.68) or use of HIT (*P*=.16) in individuals when controlled for cancer diagnosis in this hypothesis as well (Table 3).

**Table 3:** Results of univariate models controlling for race.

Predictor		Access to HIT^a^			Use of HIT	
		OR^b^ (95% CI)	*P* value	OR (95% CI)		*P* value
Cancer		0.95 (3.52-1.87)	.68	1.52 (0.84-2.7)		.16
**Race**						
	Reference (ethnic and minority groups)	1	N/A^c^	1		N/A
	White	2.47 (1.51-4.05)	>.001	1.13 (0.616-2.08)		.68

^a^HIT: health information technology.

^b^OR: odds ratio.

^c^N/A: not applicable.

#### Hypothesis 1-c

We hypothesized that there is a stronger association between older adults’ income and access to and use of HIT in individuals when controlled for cancer diagnosis.

We fit 2 logistic regression models for this hypothesis to analyze the association between income and access to and use of HIT to control for cancer diagnoses. In addition to household income, we incorporate the number of individuals living in a household to create a dichotomous poverty index. Income alone, as well as poverty, were significantly associated with access to and use of HIT. Individuals living in poverty were 79% less likely to have access to HIT (odds ratio 0.21, 95% CI 0.14-0.32; *P*<.001) and used 44% less HIT (odds ratio 0.56, 95% CI 0.33-0.93; *P*=.02) compared with individuals not living in poverty. Using income alone, compared with those with a household income of CAD $13,499 (US $14,999) or less, those with household incomes of CAD $13,500 (US $15,000) to CAD $4410 (US $49,000) were 4.4 (95% CI 2.5-7.8; *P*<.001) times more likely to access and 1.9 (95% CI 0.98-3.90; *P*=.06) times more likely to use HIT. Those with household incomes of CAD $45,000 (US $50,000) to CAD $89,100 (US $99,000) were 13.42 (95% CI 6.7-26.50; *P*<.001) times more likely to have access and 2.83 (95% CI 1.3-6.17; *P*=.008) times more likely to use HIT. Those with household incomes of above CAD $89,100 (US $99,000) were 18.7 (95% CI 7.4-46.86; *P*<.001) times more likely to have access and 4.05 (95% CI 1.57-10.47; *P*=.004) times more likely to use HIT. Hence, this hypothesis was supported in this analysis for access and use of HIT. Similar to the above 2 hypotheses, there was no significant difference in access to (*P*=.79) or use of HIT (*P*=.21) in individuals when controlled for cancer diagnosis (Table 4).

**Table 4:** Results of univariate models controlling for poverty and income.

Predictor			Access to HIT^a^					Use of HIT		
			OR^b^ (95% CI)		*P* value		OR (95% CI)			*P* value
Cancer			1.07 (0.63-1.8)		.79		1.54 (0.9-2.64)			.21
**Poverty**										
	Reference (not living in poverty)	1		N/A^c^		1			N/A	
	Living in poverty	0.21 (0.14-0.32)		<.001		0.56 (0.33-0.93)			.02	
Cancer			1.74 (0.55-5.55)		.80		1.01 (0.26-3.8)			.98
**Income (US $)**										
	Reference (0-14,999)	1		N/A		1			N/A	
	15,000-49,999	4.44 (2.5-7.8)		<.001		1.95 (0.98-3.90)			.06	
	50,000-99,000	13.42 (6.7-26.50)		<.001		2.83 (1.3-6.17)			.008	
	>99,000	18.7 (7.4-46.84)		<.001		4.05 (1.57-10.47)			.004	

^a^HIT: health information technology.

^b^OR: odds ratio.

^c^N/A: not applicable.

#### Hypothesis 1-d

We hypothesized that having more fatalistic beliefs about cancer will negatively affect access to and use of HIT in individuals when controlled for cancer diagnosis.

We used 2 logistic regression models were used to test this hypothesis. The model showed that the cancer fatalistic belief score was not associated with access to HIT (odds ratio 0.64, 95% CI 0.46-0.88; *P*=.07). However, the cancer fatalistic belief score was significantly associated with the use of HIT (odds ratio 0.64, 95% CI 0.42-0.96; *P*=.03); specifically, a 1-point increase in the fatalistic belief score was associated with a 36% decrease in the use of HIT. Hence, this hypothesis was not supported for access to HIT but was supported for the use of HIT. In line with all the aforementioned hypotheses, no significant differences were observed in access to (*P*=.75) or use of HIT (*P*=.87) among individuals after controlling for cancer diagnosis (Table 5).

**Table 5:** Results of univariate models controlling for cancer fatalistic beliefs score.

Predictor	Access to HIT^a^			Use of HIT	
	OR^b^ (95% CI)	*P* value	OR (95% CI)		*P* value
Cancer	0.78 (0.18-3.42)	.75	1.14 (0.211-22)		.87
Fatalistic beliefs score	0.64 (0.46-0.88)	.07	0.64 (0.42-0.96)		.03

^a^HIT: health information technology.

^b^OR: odds ratio.

### Aim 2: Hypothesis 2-a

To examine the relationship between access to and use of HIT and the SMD score of patient activation measures in older adults, when controlled for cancer diagnosis.

Low access to and use of the HIT will result in lower scores of the SMD of patient activation measure in individuals controlling for cancer diagnosis.

To test this hypothesis, we conducted a linear regression analysis to investigate the association between the SMD and access to and use of HIT diagnoses. Patients with access to HIT had an SMD score of 0.21 (95% CI 0.07-0.34; *P*=.003) points higher than patients who do not have access to HIT when controlling for cancer diagnosis. This finding was significant at α=.05 significance level. There was no difference in this association when controlling for cancer (*P*=.11; Table 6).

**Table 6:** Model coefficients and *P* values for the association between self-management domain (outcome) and access to health information technology (HIT) and cancer diagnosis (predictors).

Predictor	Coefficient (95% CI)	*P* value
Cancer	0.137 (−0.03 to 0.31)	.11
Access to HIT	0.21 (0.07 to 0.34)	.003

The second model indicated that the SMD score was higher by 0.28 (95% CI 0.11-0.45; *P*=.02) points for individuals who used HIT when controlling for cancer. Moreover, on average, those with cancer diagnosis reported an SMD score 0.14 (95% CI 0.006-0.278; *P*=.04) points higher than those who did not have the diagnosis when controlling for HIT use (Table 7).

**Table 7:** Model coefficients and *P* values for the association between self-management domain (outcome) and use of health information technology and cancer diagnosis (predictors).

Predictor	Coefficient (95% CI)	*P* value
Cancer	0.142 (0.006-0.278)	.04
Use	0.28 (0.11-0.45)	.002

## Discussion

### Principal Findings

The results of this analysis indicated that lower than a college education level, being a person of color, and living in poverty were significantly associated with access to HIT. Lower use of HIT was associated with lower than college education level and living in poverty. An additional finding of this analysis is the role of fatalistic beliefs in the use of HIT. Higher cancer fatalistic belief scores were significantly associated with lower use of HIT: a 1-point increase in the cancer fatalistic belief score was associated with a 36% decrease in the use of HIT. Furthermore, higher SMD scores for patient activation measures were significantly associated with higher access to and use of the HIT. Controlling for the diagnosis of cancer did not result in significant differences in the above findings, except for the SMD score and use of HIT.

Our first aim related to sociodemographic characteristics of education, race, and income and their effect on HIT use in older adults was similar to the general population use of HIT [20,37]. Being a person of color was significantly associated with lower access but not with lower use of HIT. One novel finding of our study was the role of psychological factors of fatalistic beliefs in the use of HIT. This association was not true for access to HIT. This provides evidence that fear related to the trigger factor of a cancer diagnosis may also drive engagement behaviors in treatment decision-making by having a negative effect on the HIT, which is a tool for PE [2,12]. We controlled for cancer diagnosis in this analysis as cancer patients are reported to be more actively engaged in their care [35,60]; hence, we originally hypothesized that having that diagnosis may affect the association between access to and use of HIT and education, race, household income, poverty level, and cancer fatalistic beliefs. However, we did not find no significant difference was observed.

Our second aim was to observe higher SMD scores in the SMD of patient activation in cancer patients, consistent with prior research suggesting that dealing with the health care system for an extended period may increase patient activation in patients with or before the diagnosis of cancer [32]. After adjusting for the presence of a cancer diagnosis to consider its impact on preexisting health care engagement, we observed that patients who reported access to and use of HIT had significantly higher SMD scores. This suggests that HIT may enhance self-management in older adults irrespective of whether they have a prior cancer diagnosis. Consequently, our findings may have broader implications for other chronic diseases, as HIT can play a crucial role in encouraging older adults’ active engagement in their health care.

It is also important to consider that using HIT tools is not a replacement for actual patient-provider interaction and does not diminish the human factor of health care. HIT is not able to incorporate emotional experiences, even though it may change with wider adoption of artificial intelligence in the health care universe [61]. Instead, it is a facilitator of engagement behaviors that could potentially bridge socioeconomic and communication gaps [36,62,63]. Even when patients mostly rely on medical professionals for medical knowledge regarding their diagnosis and treatment [64], those with better access to medical information technology support have a more positive attitude toward engaging in medical decision-making, as it helps in relative information seeking to relieve anxiety [65,66]. Nevertheless, face-to-face encounters or other communication methods are essential to clarify information, as test results can occasionally be misinterpreted by patients or caregivers, leading to anxiety [37].

In older adults, the use of HIT tools may not always be desired because of functional impairments, lack of self-efficacy related to lower internet-related literacy, or preference to speak to the provider [67-70]. Various other factors, such as age over 75 years [71], gender, socioeconomic status, ease of use, facilitating conditions, and individuals’ attitudes and behaviors are also reported to be associated with the preference for use of HIT in older adults [71]. However, such a preference should not be automatically assumed, given the advantages that HIT use can offer. Providers and health systems should provide equal opportunities for older adults to use various HIT tools as additional channels to engage in medical care if they are interested in using such tools [72]. In addition, different age cohorts of adults above 65 years may have different use of HIT channels, such as patient portals [36,73]. Current literature on digital health interventions often treats older adults as a uniform group, overlooking the diversity in age definitions among seniors [74]. In addition, using social media to acquire health knowledge is significantly linked to the use of HIT. The use of social media to deal with social isolation is increasing among older adults [75,76], making the incorporation of HIT highly desirable for them. This is particularly true regarding facilitating communication with health care providers and alleviating caregiving responsibilities placed on their family members [71].

Notably, psychosocial factors were equally important. Miller [77] broadly characterizes people as either “monitors” or “blunters” in the face of perceived medical threats. “Monitors” are individuals who are highly attentive and sensitized, and tend to amplify threats, whereas “blunters” avoid and minimize the same threats. Information needs may differ among individuals based on their personality styles [78,79]. HIT can act as a supplementary resource of information for individuals when older adults or their caregivers need more comprehensive details to alleviate their anxiety, especially when time constraints prevent health care providers from addressing all their questions and concerns. Hence, the preference for the use of HIT should be tailored according to the needs and preferences of a particular patient, regardless of age. Our analysis adds fatalistic beliefs about cancer as an additional psychosocial factor that may impact an individual’s preference for HIT use regardless of having a past cancer diagnosis. Cancer fatalism refers to a belief or attitude held by some individuals that cancer is an inevitable and uncontrollable disease and that there is little or nothing that can be done to prevent or treat it effectively [80]. Previous research has demonstrated that enhancing perceived confidence in overcoming health information-seeking challenges can potentially alleviate cancer fatalism [81]. HIT functions as an additional information-seeking tool, making it worthwhile for health care providers to promote its use. Importantly, provider encouragement stands out as a significant factor that can positively influence an individual’s adoption of HIT [82-84].

Limited access and use are social determinants of health [21], and older adults are one of the main groups where HIT is underutilized [85,86]. There are still opportunities to explore new directions and future applications of HIT implementation to engage older adults [27]. On the basis of our analysis, it is evident that a digital divide still exists among older adults regarding access to and use of HIT. Factors such as race, education, income, poverty, and fatalistic beliefs contribute to disparities in benefiting from HIT use at the individual level. With race, it is interesting to note that access is associated with being a person of color; however, use is not, which may suggest that if access is available, use of HIT may not be associated with being a person of color. In addition, higher SMD scores were associated with increased use of HIT, indicating HIT’s potential in promoting patient activation, leading to engagement in health care in older adults.

Given the widespread adoption of HIT, it is crucial to carefully assess interventions to ensure that they do not inadvertently exacerbate social health inequalities [87]. Future research should include diverse cohorts of older adults when designing HIT channels, such as patient portals, to ensure user-friendly interfaces tailored to their needs. Such an approach is embedded in user-centered design, which is in line with precision medicine that considers an individual’s specific sociodemographic and psychosocial factors [88]. User-centric design identifies genuine user requirements, reactions, and behaviors during design iterations, and optimizes usability and functionality [89,90]. Exploring different user-centric designs for specific diseases, such as cancer care, can be helpful for older adults who are at a higher risk of being diagnosed, and the information needs of patients are higher [91]. In the context of cancer or other chronic diseases, older adults may use web-based HIT tools in collaboration with family members or friends to make complex medical decisions [92,93]. Consequently, even if older adults have limited electronic literacy, the support of a caregiver in seeking information through HIT can still enhance their engagement in health care.

The unique needs and preferences of older adults will enable health care systems to effectively engage this population in their care and ultimately improve overall health outcomes. During chronic disease management visits for older adults, it is vital to regularly evaluate their preferences and issues related to HIT use and access. By addressing access barriers and enhancing the use of HIT among older adults, health care systems can advance health equity and diminish health disparities within this demographic.

### Limitations

The small sample size was a limitation of this study. Although HINTS has multiple cycles, questions about SMD, a precursor to PE processes, were only included in HINTS 4 cycle 3 and were not included in any other cycle. Therefore, no other cycles were combined with the data used to increase the sample size. As survey weights were included in this analysis, these results are applicable to the entire US population. However, as we were unable to implement multiple imputations for missing values, our sample size remained small. Furthermore, we dichotomized the use of HIT because we did not have sufficient observations for each of the HIT categories, such as email, text, electronic medical records, video chat, or use of an app to communicate with the provider. Therefore, we could not examine the association of the combined effect of multiple channels of HIT use on the SMD of patient activation.

Finally, only 12% of individuals with a cancer diagnosis and 23% of those without a cancer diagnosis were people of color. Along with the dramatic aging of the US population over the next several decades, there will be significant increases in racial and ethnic diversity. Thus, it is insufficient to distinguish between White individuals and people of color. Although a dichotomous race variable was significantly associated with less access to HIT, the numbers were too low, and further stratification of race could not be performed. Hence, this analysis does not portray a true picture of access to HIT across various races in the United States.

### Conclusions

Sociodemographic factors, including education, race, poverty, and fatalistic beliefs about cancer, can impact the access and use of HIT in older adults, regardless of whether they have a history of a chronic disease such as cancer. These factors can either hinder or promote technology adoption within this population. Furthermore, older adults who use HIT frequently report elevated levels of self-management, a crucial element of patient activation that drives active engagement in managing their health.

## Abbreviations

HINTS: Health Information National Trends Survey
HIT: health information technology
PE: patient engagement
SMD: self-management domain

## References

[1] Barello S, Graffigna G (2018). Tools and technologies for patients and caregivers engagement: a qualitative analysis of health professionals’ attitudes and day-to-day practice. Proceedings of the 7th International Conference on Pervasive Computing Paradigms for Mental Health, MindCare 2018.

[2] Barello S, Triberti S, Graffigna G, Libreri C, Serino S, Hibbard J, Riva G (2015). eHealth for patient engagement: a systematic review. Front Psychol.

[3] Bombard Y, Baker GR, Orlando E, Fancott C, Bhatia P, Casalino S, Onate K, Denis J-L, Pomey M-P (2018). Engaging patients to improve quality of care: a systematic review. Implement Sci.

[4] Majid U, Gagliardi A (2019). Clarifying the degrees, modes, and muddles of "meaningful" patient engagement in health services planning and designing. Patient Educ Couns.

[5] Gray TF, Nolan MT, Clayman ML, Wenzel JA (2019). The decision partner in healthcare decision-making: a concept analysis. Int J Nurs Stud.

[6] Graffigna G, Barello S (2018). Spotlight on the Patient Health Engagement model (PHE model): a psychosocial theory to understand people's meaningful engagement in their own health care. Patient Prefer Adherence.

[7] Schuster AL, Hampel H, Paskett ED, Bridges JF (2023). Rethinking patient engagement in cancer research. Patient.

[8] Grando MA, Rozenblum R, Bates D (2015). Information Technology for Patient Empowerment in Healthcare.

[9] Torous J, Michalak EE, O'Brien HL (2020). Digital health and engagement-looking behind the measures and methods. JAMA Netw Open.

[10] Tulu B, Trudel J, Strong DM, Johnson SA, Sundaresan D, Garber L (2016). Patient portals: an underused resource for improving patient engagement. Chest.

[11] Wasson JH, Forsberg HH, Lindblad S, Mazowita G, McQuillen K, Nelson EC (2012). The medium is the (health) measure: patient engagement using personal technologies. J Ambul Care Manage.

[12] Graffigna G, Barello S, Wiederhold BK, Bosio AC, Riva G (2013). Positive technology as a driver for health engagement. Stud Health Technol Inform.

[13] Ford EW, Menachemi N, Phillips MT (2006). Predicting the adoption of electronic health records by physicians: when will health care be paperless?. J Am Med Inform Assoc.

[14] What is Health IT?. HealthIT.gov.

[15] What is a patient portal?. HealthIT.gov.

[16] Moore C, Daaleman TP, Helton MR (2023). Health information technology. Chronic Illness Care: Principles and Practice.

[17] Samal L, Fu HN, Camara DS, Wang J, Bierman AS, Dorr DA (2021). Health information technology to improve care for people with multiple chronic conditions. Health Serv Res.

[18] (2023). Aging in America for advocates get the facts on healthy aging. National Council on Aging.

[19] Salmi L, Dong ZJ, Yuh B, Walker J, DesRoches CM (2020). Open notes in oncology: patient versus oncology clinician views. Cancer Cell.

[20] Anthony DL, Campos-Castillo C, Lim PS (2018). Who isn't using patient portals and why? Evidence and implications from a national sample of US adults. Health Aff (Millwood).

[21] Sieck CJ, Sheon A, Ancker JS, Castek J, Callahan B, Siefer A (2021). Digital inclusion as a social determinant of health. NPJ Digit Med.

[22] Shahrokni A, Loh KP, Wood WA (2020). Toward modernization of geriatric oncology by digital health technologies. Am Soc Clin Oncol Educ Book.

[23] Anderson M, Perrin A (2017). Technology use among seniors. Pew Research Center.

[24] Faverio M (2022). Share of those 65 and older who are tech users has grown in the past decade. Pew Research Center.

[25] Zhao YC, Zhao M, Song S (2022). Online health information seeking behaviors among older adults: systematic scoping review. J Med Internet Res.

[26] Onyeaka HK, Romero P, Healy BC, Celano CM (2021). Age differences in the use of health information technology among adults in the United States: an analysis of the health information national trends survey. J Aging Health.

[27] Harris MT, Rogers WA (2023). Developing a Healthcare Technology Acceptance Model (H-TAM) for older adults with hypertension. Ageing Soc.

[28] Managing cancer as a chronic illness. American Cancer Society.

[29] Gunn AH, Sorenson C, Greenup RA (2021). Navigating the high costs of cancer care: opportunities for patient engagement. Future Oncol.

[30] Martinez LS, Schwartz JS, Freres D, Fraze T, Hornik RC (2009). Patient-clinician information engagement increases treatment decision satisfaction among cancer patients through feeling of being informed. Patient Educ Couns.

[31] Schaeffer C (2017). Talk to Me. Oncol Issues.

[32] Bernat JK, Coa K, Blanch-Hartigan D (2017). Cancer survivors as activated patients: exploring the relationship between cancer history and patient activation. J Psychosoc Oncol.

[33] Hibbard JH, Mahoney E, Sonet E (2017). Does patient activation level affect the cancer patient journey?. Patient Educ Couns.

[34] Tolotti A, Barello S, Vignaduzzo C, Liptrott SJ, Valcarenghi D, Nania T, Sari D, Bonetti L (2022). Patient engagement in oncology practice: a qualitative study on patients' and nurses' perspectives. Int J Environ Res Public Health.

[35] (2012). Patient engagement survey results. Navigating Cancer.

[36] Zaidi M, Amante DJ, Anderson E, Ito Fukunaga M, Faro JM, Frisard C, Sadasivam RS, Lemon SC (2022). Association between patient portal use and perceived patient-centered communication among adults with cancer: cross-sectional survey study. JMIR Cancer.

[37] Coughlin S, Caplan L, Young L (2018). A review of web portal use by oncology patients. J Cancer Treatment Diagn.

[38] Clarke MA, Lyden ER, Ma J, King KM, Siahpush M, Michaud T, Idoate RE, Ramos AK (2021). Sociodemographic differences and factors affecting patient portal utilization. J Racial Ethn Health Disparities.

[39] He W, Cao L, Liu R, Wu Y, Zhang W (2022). Factors associated with internet use and health information technology use among older people with multi-morbidity in the United States: findings from the National Health Interview Survey 2018. BMC Geriatr.

[40] Age and cancer risk. National Cancer Institute.

[41] Kidd L (2021). Better patient activation is a precursor to engagement in shared decision making. Evid Based Nurs.

[42] Graffigna G, Barello S, Bonanomi A (2017). The role of Patient Health Engagement Model (PHE-model) in affecting patient activation and medication adherence: a structural equation model. PLoS One.

[43] Ratcliff CL (2019). Digital patient engagement in the precision medicine era: a framework and baseline assessment. Proceedings of the HINTS Data Users Conference.

[44] Lansky D, Glier S, Grando M, Rozenblum R, Bates D (2015). 3 Using health IT to engage patients in choosing their doctors, health plans and treatments. Information Technology for Patient Empowerment in Healthcare.

[45] Duru G, Topatan S (2023). A barrier to participation in cervical cancer screenings: fatalism. Women Health.

[46] Powe BD (1997). Cancer fatalism: spiritual perspectives. J Relig Health.

[47] Niederdeppe J, Levy AG (2007). Fatalistic beliefs about cancer prevention and three prevention behaviors. Cancer Epidemiol Biomarkers Prev.

[48] Rosenfeld M, Goldblatt H, Greenblatt-Kimron L, Cohen M (2023). "There is a god or there is no god-it is in the hands of god:" fatalistic beliefs among israeli people about cancer and their impact on behavioral outcomes. J Relig Health.

[49] Hibbard JH, Stockard J, Mahoney ER, Tusler M (2004). Development of the Patient Activation Measure (PAM): conceptualizing and measuring activation in patients and consumers. Health Serv Res.

[50] Greene J, Hibbard J, Tusler M (2005). How much do health literacy and patient activation contribute to older adults' ability to manage their health?. AARP Public Policy Institute.

[51] Moradian S, Maguire R, Liu G, Krzyzanowska MK, Butler M, Cheung C, Signorile M, Gregorio N, Ghasemi S, Howell D (2023). Promoting self-management and patient activation through eHealth: protocol for a systematic literature review and meta-analysis. JMIR Res Protoc.

[52] Hesse BW, Greenberg AJ, Peterson EB, Chou W-Y (2017). The health information national trends survey (HINTS): a resource for consumer engagement and health communication research. Inf Serv Use.

[53] Wigfall LT, Tanner AH (2018). Health literacy and health-care engagement as predictors of shared decision-making among adult information seekers in the USA: a secondary data analysis of the health information national trends survey. J Cancer Educ.

[54] Asan O, Ii FC, Nagavally S, Walker RJ, Williams JS, Ozieh MN, Egede LE (2018). Preferences for health information technologies among US adults: analysis of the health information national trends survey. J Med Internet Res.

[55] Jiang S, Hong YA (2018). Mobile-based patient-provider communication in cancer survivors: the roles of health literacy and patient activation. Psychooncology.

[56] 2013 poverty guidelines. Assistant Secretary for Planning and Evaluation.

[57] Arora NK, Hesse BW, Rimer BK, Viswanath K, Clayman ML, Croyle RT (2008). Frustrated and confused: the American public rates its cancer-related information-seeking experiences. J Gen Intern Med.

[58] Befort CA, Nazir N, Engelman K, Choi W (2013). Fatalistic cancer beliefs and information sources among rural and urban adults in the USA. J Cancer Educ.

[59] Nelson DE, Kreps GL, Hesse BW, Croyle RT, Willis G, Arora NK, Rimer BK, Viswanath KV, Weinstein N, Alden S (2004). The Health Information National Trends Survey (HINTS): development, design, and dissemination. J Health Commun.

[60] Reimer T, Gerber B (2010). Quality-of-life considerations in the treatment of early-stage breast cancer in the elderly. Drugs Aging.

[61] Koncz A (2023). Ambient intelligence and emotion AI in healthcare. The Medical Futurist.

[62] Ancker JS, Barrón Y, Rockoff ML, Hauser D, Pichardo M, Szerencsy A, Calman N (2011). Use of an electronic patient portal among disadvantaged populations. J Gen Intern Med.

[63] Grossman LV, Masterson Creber RM, Benda NC, Wright D, Vawdrey DK, Ancker JS (2019). Interventions to increase patient portal use in vulnerable populations: a systematic review. J Am Med Inform Assoc.

[64] Chua GP, Tan HK, Gandhi M (2018). Information sources and online information seeking behaviours of cancer patients in Singapore. Ecancermedicalscience.

[65] Hong YA, Hossain MM, Chou W-Y (2020). Digital interventions to facilitate patient-provider communication in cancer care: a systematic review. Psychooncology.

[66] Alpert JM, Morris BB, Thomson MD, Matin K, Brown RF (2018). Implications of patient portal transparency in oncology: qualitative interview study on the experiences of patients, oncologists, and medical informaticists. JMIR Cancer.

[67] Greysen SR, Chin Garcia C, Sudore RL, Cenzer IS, Covinsky KE (2014). Functional impairment and Internet use among older adults: implications for meaningful use of patient portals. JAMA Intern Med.

[68] Price-Haywood EG, Harden-Barrios J, Ulep R, Luo Q (2017). eHealth literacy: patient engagement in identifying strategies to encourage use of patient portals among older adults. Popul Health Manag.

[69] Hall AK, Bernhardt JM, Dodd V (2015). Older adults' use of online and offline sources of health information and constructs of reliance and self-efficacy for medical decision making. J Health Commun.

[70] Wilson J, Heinsch M, Betts D, Booth D, Kay-Lambkin F (2021). Barriers and facilitators to the use of e-health by older adults: a scoping review. BMC Public Health.

[71] Garavand A, Mohseni M, Asadi H, Etemadi M, Moradi-Joo M, Moosavi A (2016). Factors influencing the adoption of health information technologies: a systematic review. Electron Physician.

[72] Irizarry T, Shoemake J, Nilsen ML, Czaja S, Beach S, DeVito Dabbs A (2017). Patient portals as a tool for health care engagement: a mixed-method study of older adults with varying levels of health literacy and prior patient portal use. J Med Internet Res.

[73] Zaidi M, Amante DJ, Anderson E, Ito Fukunaga M, Faro JM, Frisard C, Sadasivam RS, Lemon SC (2022). Association between patient portal use and perceived patient-centered communication among adults with cancer: cross-sectional survey study. JMIR Cancer.

[74] Kokorelias KM, Nelson ML, Tang T, Steele Gray C, Ellen M, Plett D, Jarach CM, Xin Nie J, Thavorn K, Singh H (2022). Inclusion of older adults in digital health technologies to support hospital-to-home transitions: secondary analysis of a rapid review and equity-informed recommendations. JMIR Aging.

[75] Kusumota L, Diniz MA, Ribeiro RM, Silva IL, Figueira AL, Rodrigues FR, Rodrigues RA (2022). Impact of digital social media on the perception of loneliness and social isolation in older adults. Rev Lat Am Enfermagem.

[76] Kim YK, Fingerman KL (2022). Daily social media use, social ties, and emotional well-being in later life. J Soc Pers Relatsh.

[77] Miller S (1998). Monitors and blunters: different patient coping styles. Oncology NEWS international.

[78] Plamann K, McCarthy Veach P, LeRoy BS, MacFarlane IM, Petzel SV, Zierhut HA (2021). Effects of monitoring versus blunting on the public's preferences for information in a hypothetical cancer diagnosis scenario. J Genet Couns.

[79] de Rooij BH, Ezendam NP, Vos MC, Pijnenborg JM, Boll D, Kruitwagen RF, van de Poll-Franse LV (2019). Patients' information coping styles influence the benefit of a survivorship care plan in the ROGY Care Trial: new insights for tailored delivery. Cancer.

[80] Powe BD, Finnie R (2003). Cancer fatalism: the state of the science. Cancer Nurs.

[81] Paige SR, Alpert JM, Bylund CL (2021). Fatalistic cancer beliefs across generations and geographic classifications: examining the role of health information seeking challenges and confidence. J Cancer Educ.

[82] Shimoga SV, Lu YZ (2019). Role of provider encouragement on patient engagement via online portals. J Am Med Inform Assoc.

[83] Mukhopadhyay S, Basak R, Khairat S, Carney TJ (2021). Revisiting provider role in patient use of online medical records. Appl Clin Inform.

[84] Patel V, Johnson C (2018). Individuals’ use of online medical records and technology for health needs. The Office of the National Coordinator for Health Information Technology.

[85] (2023). Disparities in patient portal communication, access, and use. Health Information National Trends Survey.

[86] Tappen RM, Cooley ME, Luckmann R, Panday S (2022). Digital health information disparities in older adults: a mixed methods study. J Racial Ethn Health Disparities.

[87] Latulippe K, Hamel C, Giroux D (2017). Social health inequalities and eHealth: a literature review with qualitative synthesis of theoretical and empirical studies. J Med Internet Res.

[88] Viana JN, Edney S, Gondalia S, Mauch C, Sellak H, O'Callaghan N, Ryan JC (2021). Trends and gaps in precision health research: a scoping review. BMJ Open.

[89] De Vito Dabbs A, Myers BA, Mc Curry KR, Dunbar-Jacob J, Hawkins RP, Begey A, Dew MA (2009). User-centered design and interactive health technologies for patients. Comput Inform Nurs.

[90] Hsueh P-Y, Wetter T, Zhu X (2022). Personal Health Informatics Patient Participation in Precision Health.

[91] Reifegerste D, Rosset M, Czerwinski F, Baumann E, Gaisser A, Kludt E, Weg-Remers S (2023). Understanding the pathway of cancer information seeking: cancer information services as a supplement to information from other sources. J Cancer Educ.

[92] Oh YS (2015). Predictors of self and surrogate online health information seeking in family caregivers to cancer survivors. Soc Work Health Care.

[93] Petrillo LA, McMahan RD, Tang V, Dohan D, Sudore RL (2018). Older adult and surrogate perspectives on serious, difficult, and important medical decisions. J Am Geriatr Soc.

